# Head-Mistresses and the Nurse

**Published:** 1917-02-24

**Authors:** 


### THE MATRONS' AND SISTERS' DEPARTMENT.

### HEAD-MISTRESSES AND THE NURSE.

**[We publish these views of nurse training by an Educational Authority in the hope that they may lead to a full discussion of the questions raised.—Ed. THE HOSPITAL.]**

A circular intended to be confidential but not so marked and sent to some 200 persons is soon a *secret de Polichinelle*. There is, indeed, no reason why the circular of recommendations issued by the Head-Mistresses' Association should be kept secret. It emanates from a responsible body of women who have no private axe to grind, and most of them are interested not only in their pupils' welfare, but in all social betterment. They see that fewer of their (secondary school) pupils take up the nurse's profession, or, we would rather say, feel a vocation for it, in the original sense of that ill-used word. They suspect that a widened choice of careers is not the only cause. Reduced to their least common denominator, therefore, the Head-Mistresses' suggestions amount to a question: *Is the training of nurses rising in standard like other trainings? If not, why not? and what do you propose to do about it?* Something may depend upon the answer. If a federation of some 400 schools with, roughly, 50,000 pupils, decides that a given career is one from which these girls should be deterred, some good material will be lost and a depreciatory impression created which will be none the less real because elusive. It is, of course, true that school-leavers are too young to train at once. If they stay till eighteen there is still a five years' gap, for no wise head-mistress will advise the exhausting and unsatisfactory novitiate of this time at a children's hospital. She can give a bias to elder girls and definite counsel to ex-pupils who ask it. The girl who can go on to college will now probably proceed thence to study medicine and come into that profession on the flowing tide. The one who decides to hold to her vocation for nursing may be advised to go on learning, and if she must earn at once to avoid lessening her receptiveness by teaching. Nothing acquired will come amiss; whether domestic economy or science or the humanities. The nurse's three or four years of technical training need a sounder foundation of general education and mental discipline than they sometimes find laid. If her physique is of the strongest, the work will leave no mental or physical energy to spare during this time, not overmuch during her years of service. Now if ever is the time to gain thoughts worth thinking.

Briefly, the Head-Mistresses suggest a standard professional examination for all nurses, and to that end a standardised training. Certain hospitals should be chosen to set and carry out this training, making it probable that most others shortly, and practically all eventually, would fall into line. Meantime, on this University system external students would sit for diploma examinations. Whether these proposals were made in ignorance of the standardisation that must shortly be made by the Royal College of Nursing does not appear; at present the petitioners believe that the standard not only varies between different hospitals, but in the same hospital at different dates. It will certainly be a pity if a day ever comes on which there will be no point in saying " a So-and-so Nurse " as one speaks now of an Eton boy. But this is by the way, and the reflection is caused by the Head-Mistresses' further suggestion that in the selected hospitals uniform conditions should prevail. For the bettering of present conditions they make seven suggestions, as follows:—

1. *Eight-hour shifts, with definite time for study and lectures.*—This point will rouse much controversy. On the one hand, are even those probationers who do not need weeding out fresh enough, in certain institutions if not in others, really to profit by study?—On the other, can the lack of continuity be afforded by any serious case? If nurses can only do eight-hour shifts without undue exertion, what of, say, the first twenty-four or thirty-six hours at the private cases which some will take later? Some Colonial hospitals, at least, are enthusiasts for the eight-hour system.

2. *Regular meal hours and well-cooked food, with sufficient time to eat it.*—This with (4) *Residence in good and well-kept quarters* will rouse ire where care and expense have been lavished on the well-being of students. But some withers may be wrung, and, after all, the recommendation is merely in tune with efforts nationally made now for all servants of their country, whether soldiers, munitions-workers, or teachers.

3. *Properly planned recreation times, known beforehand by the nurses.*—Again there will be indignation, but those among the laity who have waited in Tube stations and under lamp-posts for the nurse, who arrived just after they had given her up and left, or did not arrive at all, have a cherished grievance.

5. *A library of books for reference.*—The probationer's pay is not so high that she can buy costly works, technical or other.

6. *Sufficient practice in dressings.*—Surely a preventable omission. The *Middlesex* plan of alternate days for dressings by students and nurses answers well enough, we believe.

7. *A regular rota, so that a nurse does not leave without training in all departments.*

It is, of course, easy to deprecate all such suggestions as carping and ignorant criticism. Hospitals work now, as always, under financial conditions that would not be tolerated in any business. To carry out at the same time the training of a large number of women, of whom a negligible number pay for their education, while a proportion must always be scrapped at different stages, is no light task. No doubt if hospitals are co-ordinated, or through the Royal Colleges, the present system might be replaced by apprenticeship on mediæval lines, or a paid collegiate education assisted by scholarships, or a paid training. Meantime one suggestion may be added. It is an open secret that some educated girls are deterred or thrown out by the sister or matron who does not care to ease her, necessarily and quite desirably, iron hand in any kind of glove. Hardening is one thing, brutalising is another, and a consummation not to be desired.

